# Baicalin Downregulates *Porphyromonas gingivalis* Lipopolysaccharide-Upregulated IL-6 and IL-8 Expression in Human Oral Keratinocytes by Negative Regulation of TLR Signaling

**DOI:** 10.1371/journal.pone.0051008

**Published:** 2012-12-11

**Authors:** Wei Luo, Cun-Yu Wang, Lijian Jin

**Affiliations:** 1 Faculty of Dentistry, The University of Hong Kong, Hong Kong SAR, China; 2 University of California Los Angeles, School of Dentistry, Los Angeles, California, United States of America; University of Arizona, United States of America

## Abstract

Periodontal (gum) disease is one of the main global oral health burdens and severe periodontal disease (periodontitis) is a leading cause of tooth loss in adults globally. It also increases the risk of cardiovascular disease and diabetes mellitus. *Porphyromonas gingivalis* lipopolysaccharide (LPS) is a key virulent attribute that significantly contributes to periodontal pathogenesis. Baicalin is a flavonoid from *Scutellaria radix*, an herb commonly used in traditional Chinese medicine for treating inflammatory diseases. The present study examined the modulatory effect of baicalin on *P. gingivalis* LPS-induced expression of IL-6 and IL-8 in human oral keratinocytes (HOKs). Cells were pre-treated with baicalin (0–80 µM) for 24 h, and subsequently treated with *P. gingivalis* LPS at 10 µg/ml with or without baicalin for 3 h. IL-6 and IL-8 transcripts and proteins were detected by real-time polymerase chain reaction and enzyme-linked immunosorbent assay, respectively. The expression of nuclear factor-κB (NF-κB), p38 mitogen-activated protein kinase (MAPK) and c-Jun N-terminal kinase (JNK) proteins was analyzed by western blot. A panel of genes related to toll-like receptor (TLR) signaling was examined by PCR array. We found that baicalin significantly downregulated *P. gingivalis* LPS-stimulated expression of IL-6 and IL-8, and inhibited *P. gingivalis* LPS-activated NF-κB, p38 MAPK and JNK. Furthermore, baicalin markedly downregulated *P. gingivalis* LPS-induced expression of genes associated with TLR signaling. In conclusion, the present study shows that baicalin may significantly downregulate *P. gingivalis* LPS-upregulated expression of IL-6 and IL-8 in HOKs via negative regulation of TLR signaling.

## Introduction

Periodontal disease is one of the main global oral health burdens and severe periodontal disease (periodontitis) is a major cause of tooth loss in adults globally [1]. Emerging evidence shows that it also increases the risk of some life-threating diseases like cardiovascular disease and diabetes mellitus [2]–[4]. Periodontitis is characterized by bacteria-induced, uncontrolled inflammatory destruction of tooth-supporting tissues and alveolar bone in susceptible individuals [5]. *Porphyromonas gingivalis* is a major periodontal pathogen and its lipopolysaccharide (LPS) is one of the key virulent attributes that significantly contributes to periodontal pathogenesis [6], [7]. It can stimulate the host to produce a variety of pro-inflammatory cytokines like IL-6 and IL-8, thereby involving in the initiation and progression of periodontal disease [8]–[10].

Toll-like receptors (TLRs) are a family of pattern recognition receptors (PRRs) that recognize microbial components and mediate the activation of host response [11]. Microbial LPS utilizes TLR4 to activate nuclear factor-κB (NF-κB), p38 mitogen-activated protein kinase (MAPK) and c-Jun N-terminal kinase (JNK), leading to the production of pro-inflammatory cytokines [11]. This process requires an initial recruitment of myeloid differentiation primary-response protein 88 (MyD88) to TLR4 [12]–[14]. In addition, there exists a TLR4-mediated MyD88-independent pathway that recruits toll/interleukin-1 receptor (TIR) domain-containing adaptor inducing interferon-β (TRIF) instead of recruitment of MyD88 to TLR4 in response to LPS, thereby activating the expression of interferon (IFN)-β and IFN-inducible genes like chemokine (C-X-C motif) ligand 10 (CXCL10) [15]–[18]. LPS is a TLR4 ligand and *P. gingivalis* LPS interacts with TLR4 to activate host response [19]–[21]. Nevertheless, it has been reported that *P. gingivalis* LPS could interact with TLR2 as well [22]–[24], due to the heterogeneity in lipid A structure of *P. gingivalis* LPS [8], [25], [26], and/or the contamination of LPS with some bioactive molecules like phosphorylated lipids and lipoproteins [27]–[29].

Recently, host modulatory therapy (HMT) has been proposed as a promising adjunct to conventional periodontal treatment [30], [31]. Some examples of HMT in treatment of periodontitis include subantimicrobial dose of doxycycline, lipoxins and resolvin E1 [32]–[34]. *Scutellariae radix* is an herb that has been used to treat inflammatory diseases in traditional Chinese medicine (TCM) since ancient times [35]. Baicalin is a flavonoid isolated from *Scutellaria radix* and it can suppress IL-8-induced metalloproteinase-8 (MMP-8) expression in human neutrophils [36]. In periodontal research, it has recently been shown that baicalin enables to inhibit the transcription of receptor activator of NF-κB ligand (RANKL) in human periodontal ligament cells, and reduces the loss of bone and collagens in rat models of periodontitis [37], [38]. Furthermore, baicalin may inhibit IL-1β-induced MMP-1 expression and stimulate collagen-I production in human periodontal ligament cells [39].

In the present study, we found that baicalin significantly downregulated *P. gingivalis* LPS-upregulated expression of IL-6 and IL-8. Baicalin also inhibited *P. gingivalis* LPS-induced activation of NF-κB, p38 MAPK and JNK proteins, and markedly downregulated *P. gingivalis* LPS-induced expression of genes associated with TLR signaling, such as chemokine (C-C motif) ligand 2 (CCL2), granulocyte colony-stimulating factor (G-CSF or CSF3) and CXCL10.

## Materials and Methods

### Cell Culture

HOKs isolated from normal human oral mucosa (Sciencell, CA, USA) were cultured according to the manufacturer's instructions. Prior to cell culture, culture vessels were coated with poly-L-lysine (Sigma, MO, USA) at 2 µg/cm^2^ at 37°C for 1 h. Cells were seeded at 5000 cells/cm^2^ with the oral keratinocyte medium (Sciencell). The incubation condition was set at 37°C with an atmosphere of 5% CO_2_ and 95% air. The medium was changed every two days for the first four days and daily thereafter until a monolayer was formed.

### Preparation of *P. gingivalis* LPS and Baicalin

Lyophilized LPS from *P. gingivalis* with type II *fimA* (strain code TDC60) was kindly provided by Prof. Y. Abiko (Nihon University, Japan). The LPS was prepared using the hot phenol water method [40], [41]. It was reconstituted in Dulbecco's phosphate-buffered saline (DPBS) to a concentration of 1.0 mg/ml, followed by filtration through a 0.2 µm cellulose acetate membrane filter (Millipore, MA, USA). Baicalin powder (solvent extracted with a purity >95% as tested by HPLC) was obtained from the Hong Kong Jockey Club Institute of Chinese Medicine, Hong Kong. It was dissolved in pure dimethyl sulfoxide (DMSO) (Sigma), and then diluted in DPBS to 1.0 mM and finally filtered for sterilization. Working solutions were made with fresh oral keratinocyte medium on the experiment day.

### Total RNA Extraction and cDNA Synthesis

Total RNA was extracted using the RNeasy mini kit (Qiagen, CA, USA). Briefly, cells were lysed with the buffer RLT, and the lysate was applied to an RNeasy Mini spin column. After several rounds of washes using the buffer RW1 and RPE, total RNA was bound to the column and other cell components were efficiently washed away. At the end, total RNA was eluted in RNase-free water. To avoid the contamination of genomic DNA, on-column DNase digestion was performed during RNA purification. The concentration of purified RNA was quantified by measuring its 260 nm UV absorbance on a NanoDrop spectrophotometer (Thermo, MA, USA). The integrity of purified RNA was evaluated by checking the ratio of 28 S rRNA and 18 S rRNA bands on an agarose gel. cDNA was synthesized using the Quantitect Reverse Transcription Kit (Qiagen). In brief, 1.0 µg of total RNA was pre-incubated with the gDNA Wipeout Buffer at 42°C for 2 min to remove any residual genomic DNA. The mixture was then incubated with the Quantiscript Reverse Transcriptase, Quantiscript RT Buffer and RT Primer Mix at 42°C for 30 min, followed by a termination step at 95°C for 5 min.

### Real-time Polymerase Chain Reaction (PCR)

Each real-time PCR reaction mix contained 10.0 µl of the QuantiFast SYBR green master mix (Qiagen), 1.0 µl of cDNA template (5.0 ng), 1.0 µl of forward primer (10 µM), 1.0 µl of reverse primer (10 µM) and 7.0 µl of ultra-pure water. The reaction condition was set as follows: an initial activation at 95°C for 5 min, followed by 40 cycles at 95°C for 10 s and 60°C for 30 s. The primer sequences were: for IL-6, 5′-AATCATCACTGGTCTTTTGGAG (forward), 5′-GCATTTGTGGTTGGGTCA (reverse); and for IL-8, 5′-GACATACTCCAAACCTTTCCACC (forward), 5′- AACTTCTCCACAACCCTCTGC (reverse); for β-actin (ACTB),5′-TTGGCAATGAGCGGTT (forward), 5′-AGTTGAAGGTAGTTTCGTGGAT (reverse). All the primers were designed to amplify a region that lasts 100–250 base pairs long and contains at least one intron. They had passed our in-house amplification efficiency and specificity tests prior to usage. To check for nonspecific primer binding or co-amplification of residual genomic DNA, the melting curve was analyzed after each running. To detect foreign DNA contamination, a no-template control which contained all the reagents except the cDNA template was included in each running. Raw fluorescence data were analyzed by an Excel workbook called DART-PCR which automatically calculates threshold cycles, relative quantification values and amplification efficiencies [42].

### Enzyme-linked Immunosorbent Assay (ELISA)

ELISA kits (R&D, MN, USA) were used to quantitatively determine the concentrations of IL-6 and IL-8 in culture supernatants. In brief, protein samples were pipetted into a microplate pre-coated with anti-IL-6 or anti-IL-8 antibodies and incubated at room temperature (RT) for 2 h. The plate was then washed three times with washing buffer to remove unbound samples. Subsequently, enzyme-linked polyclonal anti-IL-6 or anti-IL-8 antibodies were added and incubated at RT for 1 h. Following another three washes, a substrate solution was added and incubated at RT for 20 min. A blue color was then developed in direct proportion to the amount of the target cytokine in each well. Lastly, a stop solution was added to stop the color reaction. The absorbance was measured at 450 nm by a microplate reader (PerkinElmer, MA, USA).

### Protein Extraction

Cytoplasmic and nuclear proteins were extracted using the NE-PER Nuclear and Cytoplasmic Extraction kit (Thermo). Two reagents, the Cytoplasmic Extraction Reagents I and II, were added to cell pellets to lyse cells. The cytoplasmic proteins released were collected by centrifugation. The remaining intact nuclei were lysed with the Nuclear Extraction Reagent and the nuclear proteins released were collected by centrifugation. The concentrations of fractionated proteins were measured by the BCA protein assay kit (Thermo).

**Figure 1:**
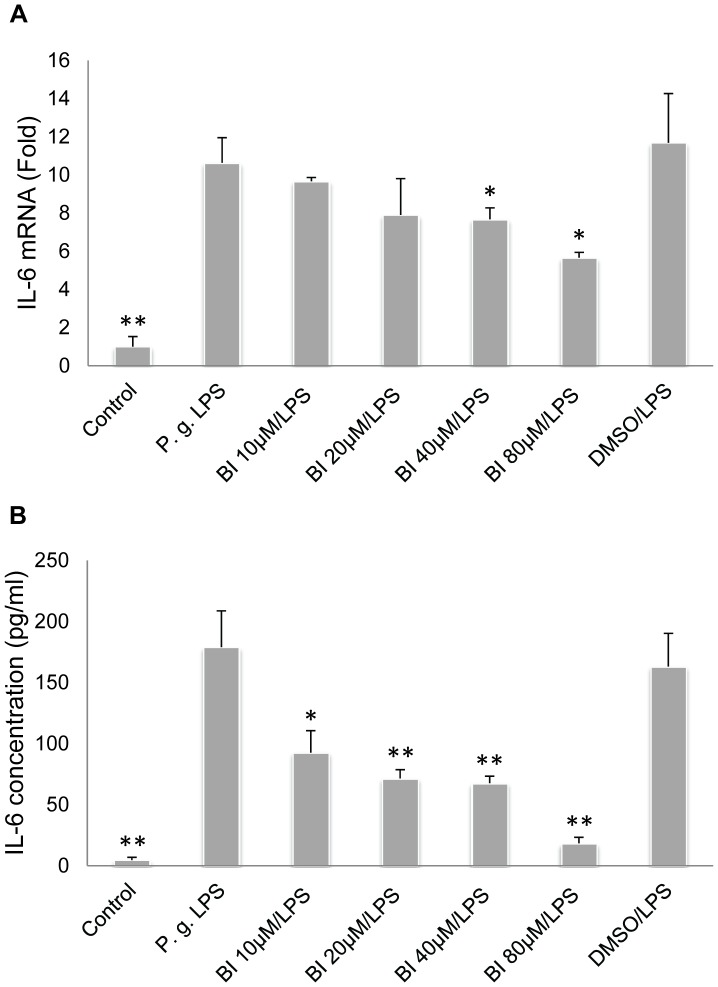
Baicalin significantly downregulates *P. gingivalis* LPS-upregulated IL-6 expression. **A**. Baicalin (BI) at 40 µM and 80 µM significantly downregulated *P. gingivalis* (*P.g.*) LPS-upregulated IL-6 mRNA expression. **B**. Baicalin at 10 µM, 20 µM, 40 µM, and 80 µM markedly downregulated *P.g.* LPS-upregulated IL-6 protein expression. Cells treated with culture media alone served as the blank control group, and those treated with *P.g.* LPS (10 µg/ml) alone represented the positive control group. Cells treated with 0.08% DMSO and *P.g.* LPS at 10 µg/ml served as the vehicle control group. Data of three independent experiments were depicted as relative fold change as compared with the blank control group (set as 1) (**A**), or presented as protein concentration (**B**). *p<0.05 and **p<0.01 as compared with the positive control group (*P.g.* LPS).

**Figure 2:**
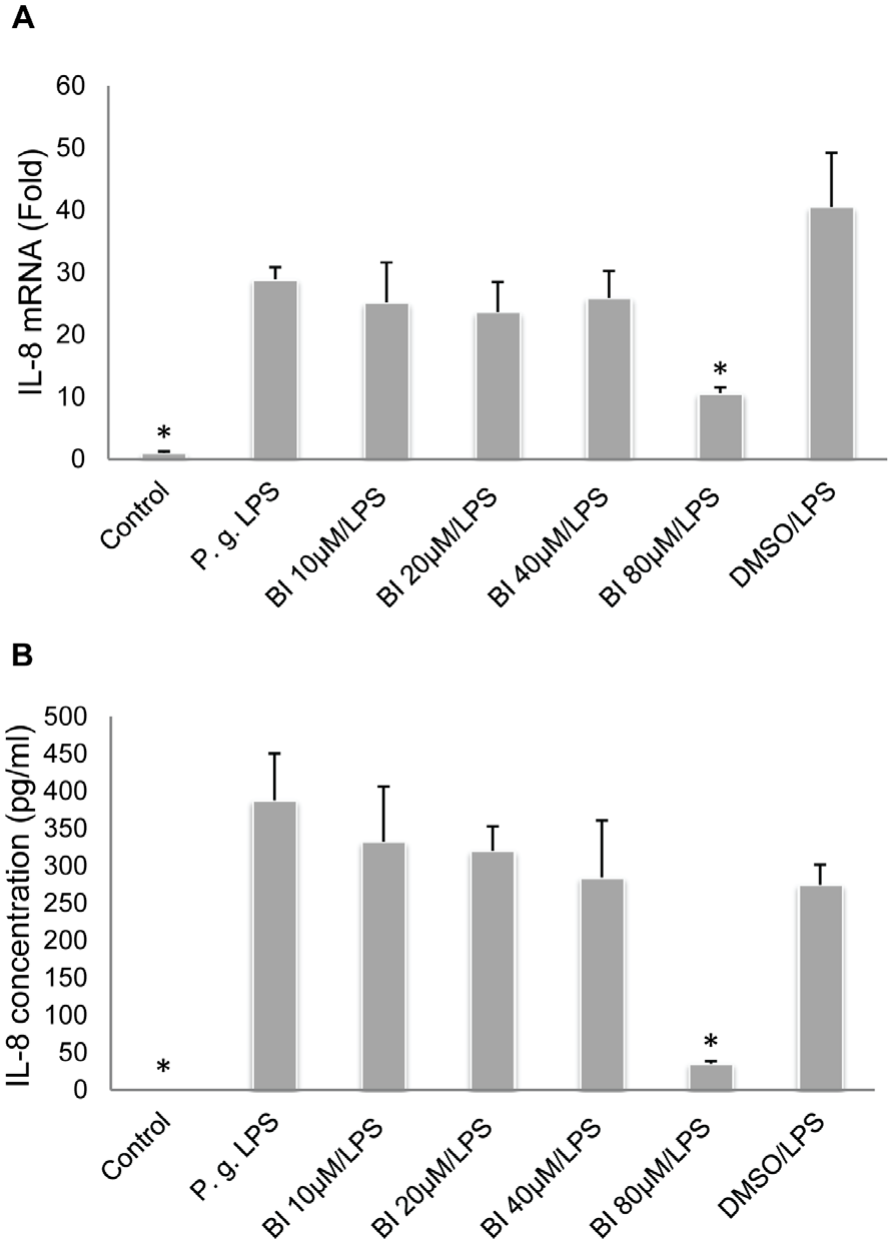
Baicalin significantly downregulates *P. gingivalis* LPS-upregulated IL-8 expression. **A**. Baicalin (BI) at 80 µM significantly downregulated *P. gingivalis* (*P.g.*) LPS-upregulated IL-8 mRNA expression. **B**. Baicalin at 80 µM significantly downregulated *P.g.* LPS-upregulated IL-8 protein expression. Cells treated with culture media alone served as the blank control group, and those treated with *P.g.* LPS (10 µg/ml) alone represented the positive control group. Cells treated with 0.08% DMSO and *P.g.* LPS at 10 µg/ml served as the vehicle control group. Data of three independent experiments were depicted as relative fold change as compared to the blank control group (set as 1) (**A**), or presented as protein concentration (**B**). *p<0.01 as compared with the positive control group (*P.g.* LPS).

### Western Blot

Protein samples were separated on 10% SDS-polyacrylamide gels by electrophoresis and subsequently transferred to polyvinylidene difluoride membranes (Roche, IN, USA) by using the Mini-PROTEAN tetra electrophoresis system and Mini Trans-Blot transfer system (Bio-rad, CA, USA). Afterwards, the membranes were incubated with the Protein-Free T20 (TBS) Blocking Buffer (Thermo) at RT for 1 h and then probed with the primary antibodies (1∶2000) at 4°C overnight with gentle agitation. On the next day, the membranes were washed and incubated with horseradish peroxidase (HRP)-conjugated secondary antibodies at RT for 1 h. They were then washed again and incubated with the SuperSignal West Pico Chemiluminescent Substrate (Thermo) for 5 min. The signals of antigen-antibody complexes were developed on X-ray films. The density of the developed bands was quantified by the ImageJ software. The rabbit monoclonal antibodies (mAbs) against human IκBα, phospho-IκBα (serine32), phospho-p38 MAPK (Thr180/Tyr182), phospho-JNK (Thr183/Tyr185) and α-tubulin were obtained from Cellsignaling (MA, USA). HRP-conjugated goat polyclonal antibodies against rabbit IgG were obtained from Thermo.

### NF-κB p65 Transcription Factor Assay

A NF-κB p65 Transcription Factor Kit (Thermo) was used to measure the level of p65 transcription factor in nuclear protein samples. It contains a 96-well plate pre-coated with a biotinylated consensus DNA sequence which only binds p65. Briefly, nuclear protein samples were added to each well with binding buffer and incubated at RT for 1 h. The plate was then washed and incubated with primary anti-p65 antibody at RT for 1 h. Following another around of washing, secondary HRP-conjugated antibodies were added to the plate and incubated at RT for 1 h. Lastly, a chemiluminescent substrate solution was added to the wells. The signal image was captured with a CCD camera and the signal intensity was measured by a multiplate reader.

**Figure 3:**
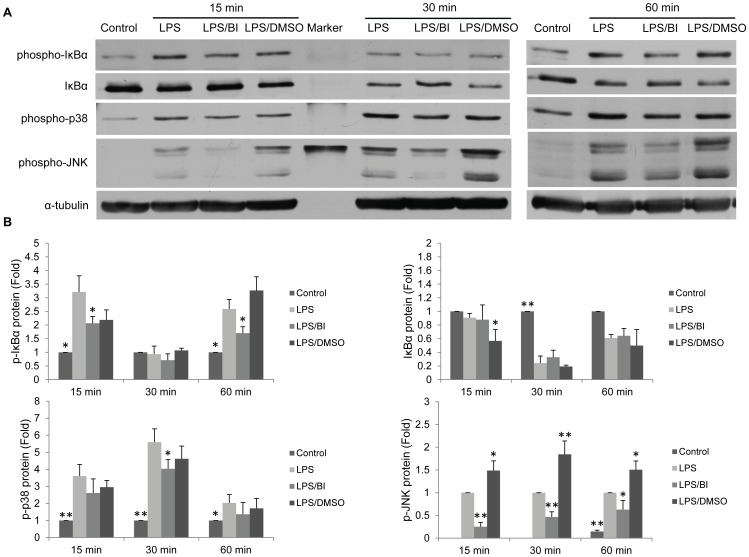
Baicalin inhibits *P. gingivalis* LPS-induced activation of NF-κB, p38 MAPK and JNK. **A**. The representative western blot experiment was performed by pooling cytoplasmic protein extracts equally from three independent experiments. 25 µg aliquots were loaded into each lane. The membrane was firstly probed with the rabbit anti*-*phospho-IκBα mAbs (1∶2000), and sequentially stripped and re-probed with rabbit anti-phospho-p38 MAPK mAbs (1∶2000), rabbit anti-phospho-JNK mAbs (1∶2000), and rabbit anti-IκBα mAbs (1∶2000). For loading control, the membrane was probed with rabbit anti-α-tubulin mAbs (1∶4000). **B**. The densitometry analysis of the signals. Cells treated with culture media alone served as the blank control group, and those treated with *P. gingivalis* (*P.g.*) LPS (10 µg/ml) alone represented the positive control group. Cells treated with *P.g.* LPS at 10 µg/ml and 0.08% DMSO served as the vehicle control group. Data of three independent experiments were depicted as relative fold change as compared with the blank control group (set as 1). For the p-JNK protein, the positive control group (LPS) was set as 1 since the signals of the blank control group at 15 min and 30 min were undetectable. *p<0.05 and **p<0.01 as compared with the respective positive control group (LPS) at each time point. BI: baicain.

### PCR Array

A panel of 89 genes associated with TLR signal transduction was investigated simultaneously using the RT^2^ Profiler™ PCR Arrays (SAbiosciences, MD, USA). RNA samples were firstly reverse transcribed into cDNA templates by the RT^2^ First Strand Kit (SAbiosciences). The diluted cDNA templates were subsequently mixed with the RT^2^ qPCR Master Mix (SAbiosciences) and H_2_O. 25 µl of the mixture were loaded into each well of the array plate which contained pre-coated specific primers. The real-time PCR was performed as follows: an initial incubation at 95°C for 10 min, then 40 cycles at 95°C for 15 s and 60°C for 1 min. Data analysis was undertaken by using the SAbiosciences web-based PCR array data analysis software.

### Statistical Analysis

All experiments were repeated three times. The data were presented as mean±SD and the statistical significance was evaluated by one way ANOVA using the SPSS 16.0 software. A *p*-value<0.05 was considered statistically significant.

**Figure 4:**
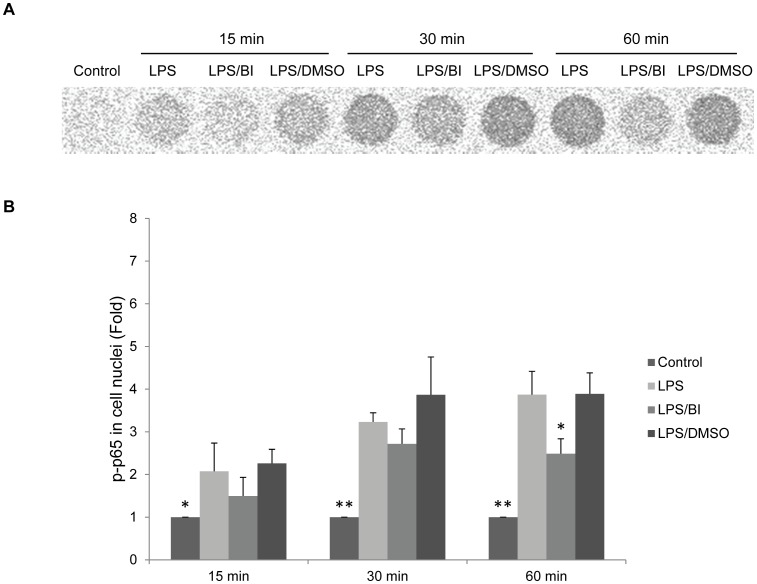
Baicalin suppresses *P. gingivalis* LPS-induced nuclear translocation of p65. **A.** The representative experiment was performed by pooling nuclear protein extracts equally from three independent experiments. 2 µg aliquots were added to each well. The assay was carried out according to the manufacturer’s instruction. **B.** The intensity analysis of the luminescent signals. Cells treated with culture media alone served as the blank control group, and those treated with *P. gingivalis* (*P.g.*) LPS (10 µg/ml) alone represented the positive control group. Cells treated with *P.g.* LPS at 10 µg/ml and 0.08% DMSO served as the vehicle control group. Data from three independent experiments were depicted as relative fold change as compared with the blank control groups (set as 1). *p<0.05 and **p<0.01 as compared with the respective positive control group (LPS) at each time point. BI: baicalin.

## Results

### Baicalin Downregulated *P. gingivalis* LPS-upregulated Expression of IL-6 and IL-8

HOKs were pre-treated with baicalin (0–80 µM) for 24 h, and subsequently treated with fresh media containing *P. gingivalis* LPS (10 µg/ml) with or without baicalin (0–80 µM) for 3 h. The culture supernatants and total RNA were collected for ELISA and real-time PCR analyses, respectively. We discovered that baicalin at 40 µM and 80 µM significantly suppressed *P. gingivalis* LPS-upregulated IL-6 mRNA expression (Fig. 1A); and baicalin at 10 µM, 20 µM, 40 µM and 80 µM significantly downregulated *P. gingivalis* LPS-upregulated IL-6 protein expression (Fig. 1B). Baicalin at 80 µM also significantly suppressed *P. gingivalis* LPS-upregulated IL-8 mRNA and protein expression (Figs. 2A & 2B). As baicalin at 80 µM contained 0.08% DMSO, the observed downregulation could have been partially caused by DMSO. To exclude this possibility, a vehicle control group was set up by treating cells firstly with 0.08% DMSO for 24 h, and then with *P. gingivalis* LPS (10 µg/ml) and 0.08% DMSO for 3 h. No DMSO-mediated inhibition on IL-6 or IL-8 expression was found (Figs. 1 and 2).

**Table 1:** The fold change in the expression of genes in baicalin/*P. gingivalis* LPS-treated cells (test) with reference to the *P. gingivalis* LPS-treated cells (control).

Wells	Genes	Fold Change	Wells	Genes	Fold Change
A01	BTK	1.04	B01	ELK1	1.37
A02	CASP8	1.11	B02	FADD	1.86
**A03**	**CCL2**	**0.13**	*B03*	*FOS*	*0.46*
A04	CD14	0.67	B04	HMGB1	1.65
*A05*	*CD80*	*2.30*	B05	HRAS	1.22
A06	CD86	1.00	B06	HSPA1A	1.66
A07	CHUK	1.21	B07	HSPD1	1.52
A08	CLEC4E	0.80	B08	IFNA1	1.44
*A09*	*CSF2*	*0.49*	*B09*	*IFNB1*	*0.41*
**A10**	**CSF3**	**0.21**	B10	IFNG	1.04
**A11**	**CXCL10**	**0.17**	B11	IKBKB	1.08
A12	EIF2AK2	0.91	B12	IL10	1.04
C01	IL12A	0.96	D01	CD180	0.60
C02	IL1A	0.77	D02	LY86	1.04
C03	IL1B	0.72	D03	LY96	1.07
C04	IL2	1.04	D04	MAP2K3	1.13
C05	IL6	0.55	D05	MAP2K4	1.19
*C06*	*IL8*	*0.34*	D06	MAP3K1	1.42
*C07*	*IRAK1*	*2.39*	D07	MAP3K7	1.40
C08	IRAK2	0.64	D08	MAP3K7IP1	1.43
C09	IRF1	1.23	D09	MAP4K4	1.08
C10	IRF3	1.14	D10	MAPK8	1.16
*C11*	*JUN*	*2.06*	D11	MAPK8IP3	1.95
C12	LTA	1.21	D12	MYD88	1.26
E01	NFKB1	0.70	F01	RIPK2	1.28
E02	NFKB2	1.54	*F02*	*SARM1*	*2.57*
E03	NFKBIA	0.81	*F03*	*SIGIRR*	*2.72*
E04	NFKBIL1	1.35	F04	ECSIT	1.98
E05	NFRKB	1.84	F05	TBK1	1.07
E06	NR2C2	1.30	F06	TICAM2	1.58
E07	PELI1	1.27	*F07*	*TIRAP*	*2.90*
E08	PPARA	1.98	*F08*	*TLR1*	*2.14*
E09	PRKRA	1.28	F09	TLR10	1.20
E10	PTGS2	0.66	F10	TLR2	0.78
E11	REL	1.32	F11	TLR3	0.88
E12	RELA	1.54	F12	TLR4	1.04
G01	TLR5	0.72	H01	B2M	0.70
*G02*	*TLR6*	*2.34*	H02	HPRT1	0.96
G03	TLR7	1.04	H03	RPL13A	1.27
G04	TLR8	1.04	H04	GAPDH	0.97
G05	TLR9	0.73	H05	ACTB	1.21
G06	TNF	0.84			
G07	TNFRSF1A	1.06			
G08	TOLLIP	1.16			
G09	TRAF6	1.35			
G10	TICAM1	0.96			
G11	UBE2N	1.20			

The genes downregulated over four folds are highlighted in bold, and those up or downregulated two to four folds are highlighted in italics.

### Baicalin Displayed Inhibitory Effect on *P. gingivalis* LPS-induced Activation of NF-κB, p38 MAPK and JNK

In resting cells, inactive NF-κB (p65/p50) is retained in the cytoplasm by an inhibitory protein called IκBα [43]. Upon stimulation, IκBα is ubiquitinated and degraded by 26 S proteasome, resulting in the translocation of NF-κB to the nucleus where it binds to the target genes and initiates gene transcription [43]. As NF-κB plays a central role in *P. gingivalis* LPS-mediated cell response and the expression of IL-6 and IL-8 is dependent on NF-κB signaling [44], we were interested to exam whether baicalin could have any inhibitory effects on *P. gingivalis* LPS-activated NF-κB. Cells were pre-treated with baicalin (80 µM) for 24 h, and thereafter treated with fresh media containing *P. gingivalis* LPS (10 µg/ml) with or without baicalin (80 µM) for 15, 30, and 60 min. A vehicle control group was set up by treating cells firstly with 0.08% DMSO for 24 h, and then with *P. gingivalis* LPS (10 µg/ml) and 0.08% DMSO for 15, 30, and 60 min. As shown in Fig. 3, baicalin significantly inhibited to different extents *P. gingivalis* LPS-induced phosphorylation of IκBα, p38 MAPK and JNK which act as the downstream of TLR2/4 signaling pathways [45].

### Baicalin Suppressed *P. gingivalis* LPS-induced Nuclear Translocation of p65

The effect of baicalin on *P. gingivalis* LPS-induced nuclear translocation of p65 was examined by using a p65 transcription factor kit. Cells were pre-treated with baicalin (80 µM) for 24 h, and then treated with fresh media containing *P. gingivalis* LPS (10 µg/ml) with or without baicalin (80 µM) for 15, 30, and 60 min. Compared with *P. gingivalis* LPS-treated samples, baicalin succeeded to suppress the amount of translocated p65 in the nuclear protein extracts at 60 min (Fig. 4).

### Baicalin Modulated *P. gingivalis* LPS-induced Expression of Genes Associated with TLR Signaling

Lastly, a PCR array assay was undertaken to profile the expression of genes associated with TLR signaling. Cells were pre-treated with baicalin (80 µM) or culture media for 24 h, and then treated with fresh media containing *P. gingivalis* LPS (10 µg/ml) with or without baicalin (80 µM) for 3 h. The total RNA was purified and reverse transcribed into cDNA templates. The templates used in PCR array were pooled equally from three independent experiments. Compared with the *P. gingivalis* LPS-treated cells, the expression of CCL2, CSF2, CSF3, CXCL10, IL-8, V-fos FBJ murine osteosarcoma viral oncogene homolog (FOS) and interferon, beta 1, fibroblast (IFNB1) was significantly downregulated over two folds in baicalin/*P. gingivalis* LPS treated cells (Table 1). Notably, CCL2, CSF3 and CXCL10 were markedly downregulated over four folds. On the other hand, other genes including cluster of differentiation 80 (CD80), interleukin-1 receptor-associated kinase 1 (IRAK1), jun proto-oncogene (JUN), TLR6, Ubiquitin-conjugating enzyme E2 variant 1 (UBE2V1), sterile α and TIR motif–containing 1 (SARM1), single Ig IL-1-related receptor (SIGIRR), TIR domain containing adaptor protein (TIRAP) and TLR1 were significantly upregulated over two folds in baicalin/LPS-treated cells (Table 1).

## Discussion

Periodontal disease results essentially from the consequence of a disrupted immuno-inflammatory homeostasis of bacteria-host interactions [5]. In susceptible individuals, when host response fails to limit and resolve early infection timely, cytokine expression may become dysregulated and destructive to tissues [46], [47]. As IL-6 is a stimulator of bone resorption and IL-8 is a potent neutrophil chemoattractant and activator [48], [49], prolonged and excessive production of these pro-inflammatory cytokines could contribute to periodontal tissue damage. Our present study shows that baicalin could significantly downregulate *P. gingivalis* LPS-upregulated production of IL-6 and IL-8 in HOKs. This observation goes in line with the concept of host modulatory therapy, suggesting that baicalin could potentially be used for modulation of host response in treatment of periodontal disease.

The present study also reveals that baicalin may inhibit *P. gingivalis* LPS-induced activation of NF-κB, p38 MAPK and JNK. Due to their involvements in a variety of human diseases, NF-κB, p38 MAPK and JNK have become therapeutic targets and several NF-κB inhibitors have been discovered, such as sulindac [50], IKK inhibitor [51], [52] and resveratrol [53]. It has been shown that SD828, a p38 MAPK antagonist, could suppress LPS-induced alveolar bone loss in periodontitis rats [54], and JNK inhibitors like CEP-1347 and AS601245 exhibit protective effects on neurons [55], [56]. In the present study, the exact mechanism of baicalin-induced inhibition of *P. gingivalis* LPS-upregulated expression of IL-6 and IL-8 in HOKs remains to be further elucidated. While it could be speculated that the inhibition observed could have been exerted directly on IKK, p38 MAPK and JNK; or on the upstream kinases such as transforming-growth-factor-β-activated kinase 1 (TAK1) (kinase of IKK and p38/JNK MAPK) [57]–[59], interleukin-1 receptor-associated kinase 1 (IRAK1) (kinase of TAK1), or IRAK4 (kinase of IRAK1) [60]–[62].

According to the PCR array results, CCL2 [63], CSF3 [64], [65] and CXCL10 [66] were greatly downregulated over four folds by baicalin treatment. The transcription of CCL2 and CSF3 is regulated by NF-κB [67], [68]. In response to LPS, CXCL10 is induced in a TLR4-mediated MyD88-independent pathway [15], [69], [70]. The exact reasons that baicalin could downregulate both LPS-induced MyD88-dependent and MyD88-independent genes remain unclear. The possible mechanisms are as follows: i) baicalin might enable to interfere with the binding of *P. gingivalis* LPS to TLR4; ii) it could inhibit multiple downstream kinases of TLR4 signaling, such as IKK, TAK1 and TANK-binding kinase 1 (TBK1) (kinase of IRF3) [71]; iii) as the optimal transcription of CXCL10 requires a coordinated binding of activated IRF3 and NF-κB to the promoter region [15], [69], baicalin-mediated inhibition of NF-κB could have interfered with the expression of CXCL10. Further study is warranted to clarify these points.

Over the last three decades, the growing knowledge of periodontal pathogenesis has appreciated the crucial role of host response in the initiation and development of periodontal disease. Recently, TLR signaling has become an attractive target of host modulation therapy due to its central role in activating immuno-imflammatory response in the development of periodontitis [72]. To date, a number of negative regulatory strategies for over-activated TLR signaling have been proposed, such as natural/synthetic antagonists [73], [74], BB-loop peptides [75], miRNA [76] and kinase inhibitors [77]. Here, we report for the first time that baicalin can significantly downregulate *P. gingivalis* LPS-upregulated IL-6 and IL-8 expression in HOKs, through negative regulation of TLR signaling. Based on these findings, baicalin may potentially serve as a host response modulator in the control of periodontal disease by negative regulation of TLR signaling. Further clinical study is warranted to investigate the effectiveness of baicalin as a potential adjunct in treatment of patients with inflammatory diseases like periodontal disease.
